# Pharmacological and mechanistic study of PS1, a Pdia4 inhibitor, in β-cell pathogenesis and diabetes in *db/db* mice

**DOI:** 10.1007/s00018-022-04677-5

**Published:** 2023-03-19

**Authors:** Hui-Ju Tseng, Wen-Chu Chen, Tien-Fen Kuo, Greta Yang, Ching-Shan Feng, Hui-Ming Chen, Tzung-Yan Chen, Tsung-Han Lee, Wen-Chin Yang, Keng-Chang Tsai, Wei-Jan Huang

**Affiliations:** 1 Ph.D. Program in Drug Discovery and Development Industry, College of Pharmacy, Taipei Medical University, Taipei City, Taiwan; 2 Agricultural Biotechnology Research Center, Academia Sinica, 128, Academia Rd. Section 2, Nankang, Taipei City, Taiwan; 3 Department of Aquaculture, National Taiwan Ocean University, Keelung City, Taiwan; 4 Translational Biomedical Research Center, Academia Sinica, Taipei City, Taiwan; 5 Department of Life Sciences, National Chung Hsing University, Taichung City, Taiwan; 6 National Research Institute of Chinese Medicine, Ministry of Health and Welfare, Taipei City, Taiwan; 7 Graduate Institute of Integrated Medicine, China Medical University, Taichung, Taiwan; 8 Graduate Institute of Pharmacognosy, College of Pharmacy, Taipei Medical University, Taipei City, Taiwan; 9 Program for the Clinical Drug Discovery From Botanical Herbs, College of Pharmacy, Taipei Medical University, Taipei City, Taiwan; 10 School of Pharmacy, National Defense Medical Center, Taipei City, Taiwan; 11 Ph.D. Program in Medical Biotechnology, College of Medical Science and Technology, Taipei Medical University, Taipei City, Taiwan

**Keywords:** Protein disulfide isomerase a4, Hyperglycemia, β-Cell death, β-Cell dysfunction, Oxidative stress, Cure and small-molecule drug

## Abstract

Pdia4 has been characterized as a key protein that positively regulates β-cell failure and diabetes via ROS regulation. Here, we investigated the function and mechanism of PS1, a Pdia4 inhibitor, in β-cells and diabetes. We found that PS1 had an IC_50_ of 4 μM for Pdia4. Furthermore, PS1 alone and in combination with metformin significantly reversed diabetes in *db/db* mice, 6 to 7 mice per group, as evidenced by blood glucose, glycosylated hemoglobin A1c (Hb_A1c_), glucose tolerance test, diabetic incidence, survival and longevity (*P* < 0.05 or less). Accordingly, PS1 reduced cell death and dysfunction in the pancreatic β-islets of *db/db* mice as exemplified by serum insulin, serum c-peptide, reactive oxygen species (ROS), islet atrophy, and homeostatic model assessment (HOMA) indices (*P* < 0.05 or less). Moreover, PS1 decreased cell death in the β-islets of *db/db* mice. Mechanistic studies showed that PS1 significantly increased cell survival and insulin secretion in Min6 cells in response to high glucose (*P* < 0.05 or less). This increase could be attributed to a reduction in ROS production and the activity of electron transport chain complex 1 (ETC C1) and Nox in Min6 cells by PS1. Further, we found that PS1 inhibited the enzymatic activity of Pdia4 and mitigated the interaction between Pdia4 and Ndufs3 or p22 in Min6 cells (*P* < 0.01 or less). Taken together, this work demonstrates that PS1 negatively regulated β-cell pathogenesis and diabetes via reduction of ROS production involving the Pdia4/Ndufs3 and Pdia4/p22 cascades.

**Supplementary Information:**

The online version contains supplementary material available at 10.1007/s00018-022-04677-5.

## Introduction

The International Federation of Diabetes estimated that in 2021 there were 537 million people living with diabetes worldwide and 5 million lives were lost due to the disease [1]. One of the characterizing traits of diabetes is the failure of functional β-cells to modulate insulin secretion to offset increasing insulin resistance, driving disease development [2]. Unfortunately, no anti-diabetes drugs are available in the clinic for preserving β-cells. Beta-cell failure is known to be important in diabetes development [3], and preservation of functional β-cells can reverse the outcome of clinical diabetes [4, 5]. Thus, the search for new genes implicated in β-cell failure can assist in developing therapeutic approaches to cure diabetes.

Despite some progress on β-cell failure, the molecular mechanism that orchestrates β-cell number and function is poorly studied. Diabetogenic stimuli always cause endoplasmic reticulum (ER) stress and oxidative stress [6, 7]. Consequently, exuberant reactive oxygen species (ROS) result in β-cell demise and dysfunction [8, 9] and peripheral insulin resistance [10] in animals and humans. Besides, β-cells are known to be susceptible to ROS because they possess fewer anti-oxidant proteins than the other cell types [2]. The mitochondrial electron transport chain (ETC), NADPH oxidase (Nox), and ER oxidoreductin 1 are common machineries for ROS generation [11–13].

Some potential molecular targets of β-cell failure have been identified including BACH2 [14], MST1 [15], GSK3 [16], PDX1 [16], P2RY1 [17], PHLPP [18], Pdia4 [2], etc. Among them, Pdia4 was the only target whose deficiency could reverse diabetes [2]. Pdia4 is a molecular chaperone with 3 GCHC motifs in the Pdi family. Pdia4 has been reported to be mainly expressed in β-cells, and its expression was up-modulated in β-cells and sera of rodents by nutrient overload [2]. Furthermore, data obtained from Pdia4 knockout and transgenic mice showed that Pdia4 promoted β-cell failure, including cell death and dysfunction, and diabetes via up-regulation of ROS production as indicated by fasting blood glucose (FBG), postprandial blood glucose (PBG), glycosylated hemoglobin A1c (Hb_A1c_), glucose tolerance test (GTT), islet architecture, diabetic incidence, and homeostatic model assessment for β-cell function and insulin resistance (HOMA) indices. Mechanistically speaking, Pdia4 was found to augment ROS production in β-cells via its interplay and activation of Ndufs3 and p22 in the ETC complex 1 (ETC C1) and Nox pathways, respectively [2]. This seminal publication identified Pdia4 as a novel therapeutic target of β-cell pathogenesis and diabetes as a result of ROS dysregulation [2].

PS1 has been approved as an investigational new drug for diabetes by the Food and Drug Administration of the United States (https://clinicaltrials.gov/ct2/show/NCT05176210?term=PS1&draw=2&rank=1). We hypothesized that PS1, a drug candidate that inhibits Pdia4, could reverse β-cell pathogenesis and diabetes in *db*/*db* mice. In the current investigation, we studied the pharmacological effect and mechanism of PS1 at the molecular, cellular and animal levels.

## Materials and methods

### Cells, plasmids, and reagents

A murine β-cell line, Min6 cells, and pancreatic islets isolated from B6 mice were grown in DMEM medium (Sigma D5648) supplemented with 20% FBS, 3.4 mg/mL NaHCO_3_, 75 μg/mL penicillin, 50 μg/mL streptomycin, and glucose at the indicated dosages. The cells were grown at 37 °C in a 5% CO_2_ incubator. Expression plasmids, Flag-Pdia4, Myc/Flag-tagged Ndufs3, and Myc/Flag-tagged p22, were constructed and transfected as published previously [2]. PS1 with a molecular formula of C_12_H_11_NO_5_ (Pharmasaga, Taipei, Taiwan), sitagliptin (STG, Merck, Kenilworth, NJ), metformin (Merck) and optimal cutting temperature (OCT, Thermo Fisher, Waltham, MA) were obtained. Diaminobenzidine tetrahydrochloride (DAB), NADPH, histopaque-1077, BSA, glucose, PBS, trypsin, and palmitate were bought from Sigma (St Louis, MO). Propidium iodide (PI), dihydroethidium (DHE), DAPI, chloromethyl-2′,7′-dichlorodihydrofluorescein diacetate (CM-H2DCFDA), CellROX, MitoSOX, MitoGreen, and Hochest 33,342 were bought from Molecular Probes (Eugene, OR). Elite glucometers and chips were purchased from Bayer (Germany). Enzyme-linked immunosorbent assay (ELISA) kits for insulin and c-peptide were purchased from Mercodia (Uppsala, Sweden) and Crystal Chem (Elk Grove Village, IL). Lucigenin, HEPES, and PBS were purchased from Roche (Switzerland). Antibodies against Ki67 (MIB-1, Santa Cruz Biotech, Santa Cruz, CA) and insulin (H-86, Santa Cruz Biotech) and terminal deoxynucleotidyl transferase dUTP nick end labeling (TUNEL) kits (Chemicon, Temecula, CA) were purchased.

### Drug administration and measurement of metabolic parameters

PBS vehicle (CTR), PS1 (0.8 mg/kg, 2.5 mg/kg, and 7.5 mg/kg) alone and in combination (2.5 mg/kg PS1 with 60 mg/kg metformin), and two therapeutic positive controls, 15 mg/kg STG alone and in combination (15 mg/kg STG plus 60 mg/kg metformin) were given orally to female *db/db* mice, a mouse model of type 2 diabetes, once a day from 8 to 24 weeks of age. The number of mice in each group was 9 females per group except for the group of PS1 at 2.5 and 7.5 mg/kg, where 10 females were used. By 18 weeks of age, 3 mice in each group were removed for histochemical analysis. The drugs were withdrawn from each group of mice from 25 weeks of age to death. Food intake, water consumption, body weight, FBG, PBG, Hb_A1c_, diabetic incidence, HOMA-β and HOMA-IR, GTT, serum insulin, serum c-peptide, and serum ROS of the mice were monitored at the time points shown. To measure FBG and PBG, the mice were fasted overnight and bled for blood samples. The mice had free access to food for 2 h. After 30 min, they were bled for blood samples. The levels of blood glucose from fasted and starved mice were measured using an Elite glucometer (Bayer, Germany). Mice with FBG or PBG over 126 or 200 mg/dL, respectively, for two consecutive measurements were considered diabetic. Diabetic incidence was calculated based on FBG and PBG. For GTT, *db/db* mice at 8, 16, 24 weeks were denied access to chow for 16 h. The mice were administered an intraperitoneal injection of 1 g/kg glucose. Blood samples were collected from tail veins at 0, 30, 60, 120 and 180 min after glucose injection. The levels of blood glucose were measured using an Elite glucometer. HOMA-β [20 × fasting insulin (mU/mL)/(fasting glucose (mmol/L)—3.5] and HOMA-IR [fasting glucose (mmol/L) × fasting insulin (μU/mL)/22.5] indices were calculated. The level of serum c-peptide, serum insulin, and Hb_A1c_ in mouse blood samples was determined as published previously [2]. The mouse sera were incubated with lucigenin [19], and ROS was assessed using a SpectraMax i3x reader (Molecular Devices, San Jose, CA). All animals were maintained at 21–23 °C with 12 h light-12 h dark cycles in the institutional animal facility and handled according to the Academia Sinica Institutional Animal Care and Utilization Committee (11-03-158).

### Flow cytometric analysis

Min6 cells were pre-incubated with complete DMEM medium containing PBS (CTR and HG), STG (10 μg/mL) and PS1 (0.2, 1, and 5 μg/mL) for 2 h. The cells were then incubated with DMEM medium, and the medium was supplemented with 30 mM glucose (HG, STG, and PS1) for an additional 2 h. After washing, the cells were stained with PI (1 μg/mL) for 5 min and analyzed using a LSRII analyzer. FlowJo software was used to analyze the cytometric data.

### Pdia4 assays

As published [2], recombinant Pdia4 was incubated with PBS vehicle and PS1 in the presence of insulin substrates at 25 °C for 30 min. Following stop solution and detection reagents, the turbidity of each sample was measured using a Biotek Cytation 5 reader at 595 nm and, subsequently, Pdia4 activity was determined. Alternatively, Pdia4 was precipitated from the pancreata of *db/db* mice given PBS and drugs at the indicated dosages using anti-Pdia4 antibody.

### Immunoblotting assays

Min6 cells were transiently transfected with the construct expressing Flag-Pdia4 and that encoding Myc/Flag-tagged Ndufs3 (B) or Myc/Flag-tagged p22 using a TransIT-LT1 Transfection kit (Mirus Bio). Following 24 h culture, the cells were treated with PBS and PS1 (5 μM) for 0.5 h. Following cell lysis, total lysates and anti-Pdia4 precipitates were subjected to immunoblot analysis with anti-Flag antibody.

### Measurement of ROS and the activity of ETC C1 and Nox [2]

Min6 cells were treated with medium, 16.7 mM glucose, and a combination of 16.7 mM glucose with STG (10 μg/mL) and PS1 at 0.2, 1, and 5 μg/mL for 30 min. The cells were incubated with Hoechst 33,342, a nuclear dye, plus CM-H_2_DCFDA, a dye for cytosolic ROS, and MitoGreen, a mitochondrial tracker, plus MitoSOX, a dye for mitochondrial ROS, and analyzed using confocal microscopy. To measure the ETC C1 and Nox activity, membrane and mitochondrial fractions of the cells were individually extracted (protein extraction kits, Abcam, UK) and tested for the activity of Nox and ETC C1, respectively, as previously described [2].

### Insulin quantification

Min6 cells were pre-treated in complete DMEM with 30 mM glucose at 37 °C for 2 h. The cells were washed extensively and stimulated with medium containing high glucose (16.7 mM), STG (10 µg/mL) and PS1 (0.2 µg/mL, 1 µg/mL, and 5 µg/mL) at 37 °C for 30 min. Their supernatants were collected. Alternatively, Min6 cells were stimulated with DMEM medium or the medium KCl (30 mM), STG (10 µg/mL) and PS1 (0.2 µg/mL, 1 µg/mL, and 5 µg/mL) at 37 °C for 30 min. The supernatants were collected. The supernatants from Min6 cells or mouse serum were measured for insulin ELISA assays according to the manufacturer’s protocol (Mercodia, Uppsala, Sweden).

### Immunohistochemical (IHC) analysis

After sacrifice, the pancreata from 3 mice per group were frozen in OCT medium and cryosectioned. The sections were stained with DHE for ROS content. To stain for insulin, the pancreatic sections were fixed and incubated with anti-insulin antibody and developed with DAB. To assess β-cell proliferation, the pancreatic sections were stained and developed with anti-Ki67 kits (BD Biosciences, San Jose, CA). For cell death detection, the pancreatic sections underwent TUNEL assays (Chemicon, Temecula, CA). The above slides were imaged and analyzed using the AxioVision program (Carl Zeiss). To quantify cell proliferation and death in β-cells, the Ki67-positive or TUNEL-positive cells were counted under a microscope.

### Statistics

Data from three or more independent experiments are expressed as mean ± standard deviation (SD). Nonparametric tests and log rank were used to determine if there are any statistical differences between groups. *P* (*) < 0.05; *P* (**) < 0.01 and *P* (***) < 0.001 were considered statistically significant. The number of mice (*n*) is shown in parentheses.

## Results

### PS1 alone and in combination with metformin can reduce diabetes and increase survival and life span in *db/db* mice

**Fig. 1:**
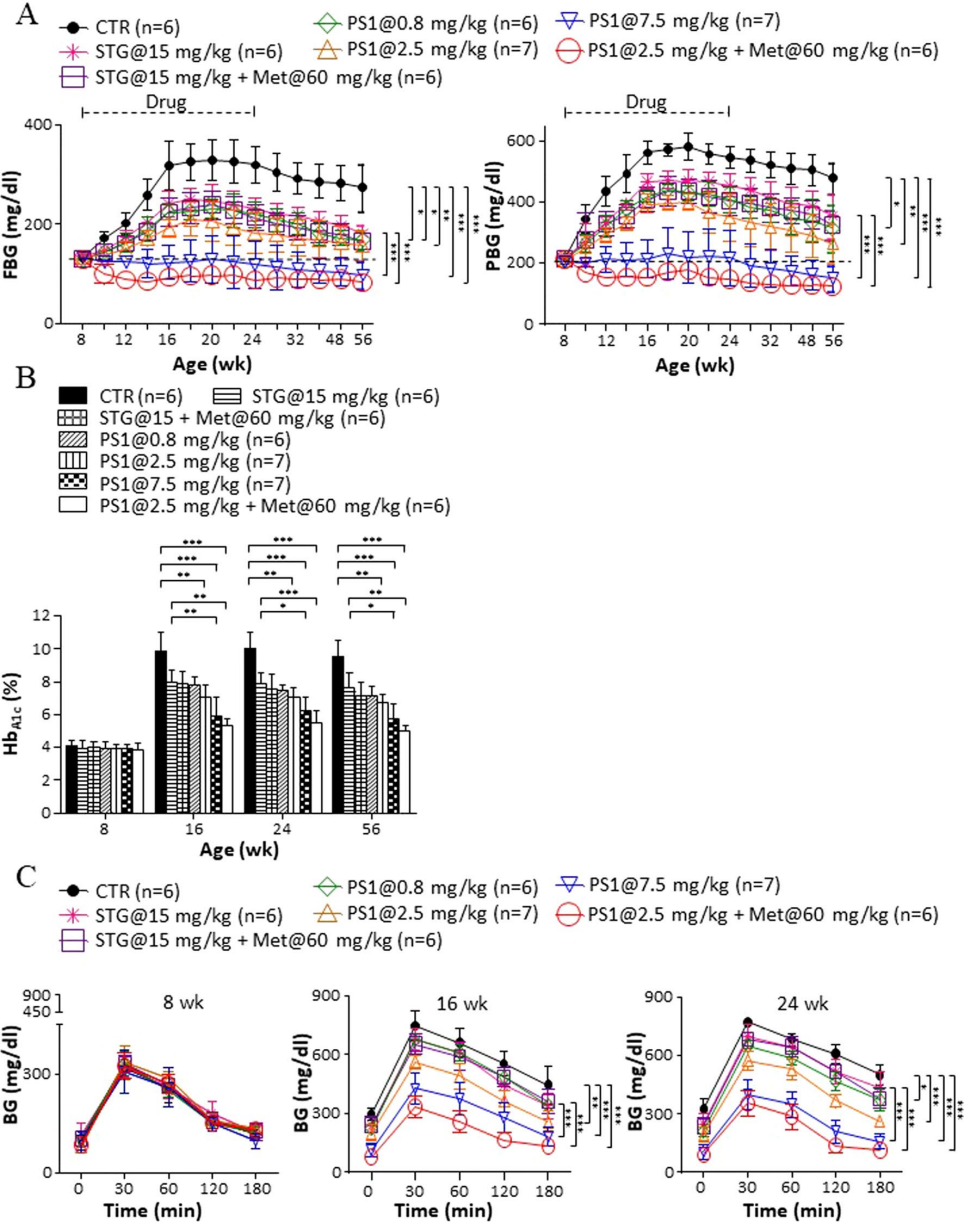
Beneficial effects of PS1 on diabetes development in *db/db* mice. **A** Female *db/db* mice that had new-onset diabetes were grouped and treated with PBS (CTR), STG (15 mg/kg), a combination of STG (15 mg/kg) and metformin (Met, 60 mg/kg), PS1 (0.8 mg/kg, 2.5 mg/kg, and 7.5 mg/kg), and a combination of PS1 (2.5 mg/kg) and metformin (60 mg/kg) from 8 to 56 weeks of age. Their fasting blood glucose (FBG) and postprandial blood glucose (PBG) levels were found using a glucometer at the indicated ages. **B** Hb_A1c_ of the mice **A** was monitored. **C** GTT of the mice **A** was monitored by the aged of 8, 16 and 24 weeks. Mouse number (*n*) is shown in parentheses. *P* (*) < 0.05, *P* (**) < 0.01 and *P* (***) < 0.001 were considered statistically significant

PS1 was rationally developed as a Pdia4 small-molecule inhibitor using a combination of a molecular docking strategy and total chemical synthesis (Figure S1A). Furthermore, PS1 had a half maximal inhibitory concentration (IC_50_) value of 4 μM for Pdia4. These data prompted us to assess the anti-diabetic effect of PS1 in *db/db* mice in new-onset diabetes (Figure S1B). First, we assessed the glucose-reduction caused by PS1 in diabetic *db/db* mice. As anticipated, *db/db* mice had manifested diabetes by 8 weeks and this disease lasted for their life time (CTR, Fig. 1A). In contrast, STG is a commercial DPP4 inhibitor for diabetes. Sixteen-week treatment with STG, a positive control of monotherapy, and 16-week treatment with STG plus metformin, a positive control of combinational therapy, modestly lowered FBG and PBG in diabetic *db/db* mice (STG@15 mg/kg versus STG@15 mg/kg + Met@60 mg/kg, Fig. 1A). Of note, PS1 dose-dependently reduced diabetes in diabetic *db/db* mice as evidenced by FBG (PS1, left, Fig. 1A) and PBG (PS1, right, Fig. 1A). A combination of PS1 and metformin had slightly better glucose-lowering effects than PS1 alone (FBG and PBG, PS1@2.5 + Met@60 mg/kg, Fig. 1A).

Hb_A1c_ is known as a reliable marker of chronic glycemia. Therefore, we next checked the effects of PS1 on Hb_A1c_ in diabetic *db/db* mice. Each group of *db/db* mice, aged 8 weeks, had an Hb_A1c_ value of 4% (8 weeks, Fig. 1B). This value increased to 10% in control *db/db* mice aged 16 weeks and beyond (CTR, 16–56 weeks, Fig. 1B). In contrast, *db/db* mice treated with STG per se and in combination with metformin reduced the Hb_A1c_ value to 8.3–7.7% in age-matched *db/db* mice (STG@15 mg/kg versus STG@15 mg/kg + Met@60 mg/kg, Fig. 1B). Furthermore, PS1 dose-dependently reduced the Hb_A1c_ value to 7.9–5.9% in age-matched *db/db* mice (PS1, Fig. 1B). The *db/db* mice given a combination of PS1 and metformin had an Hb_A1c_ value of 5.3%, which was slightly lower than the Hb_A1c_ value in the age-matched mice treated with PS1 alone (PS1@2.5 + Met@60 mg/kg versus PS1, Fig. 1B).

Next, GTT was measured in each group of *db/db* mice aged 8, 16 and 24 weeks. No difference was observed in the GTT of each group of mice at the age of 8 weeks (left, Fig. 1C). However, STG per se and in combination with metformin significantly improved GTT compared to PBS vehicle in *db/db* mice aged 16 weeks (STG@15 mg/kg versus STG@15 mg/kg + Met@60 mg/kg, middle, Fig. 1C). PS1 alone and in combination with metformin further improved GTT in 16-week-old *db/db* mice in a dose-dependent fashion (PS1 at 0.8, 2.5 and 7.5 mg/kg versus PS1@2.5 mg/kg + Met@60 mg/kg, middle, Fig. 1C). A combination of PS1 and metformin improved GTT more than PS1 alone in 16-week-old *db/db* mice (PS1 at 0.8, 2.5 and 7.5 mg/kg versus PS1@2.5 mg/kg + Met@60 mg/kg, middle, Fig. 1C). By 24 weeks of age, PS1 alone and in combination with metformin for 16 weeks improved GTT in *db/db* mice more than 8-week treatment with the same drugs (right, Fig. 1C).

**Fig. 2:**
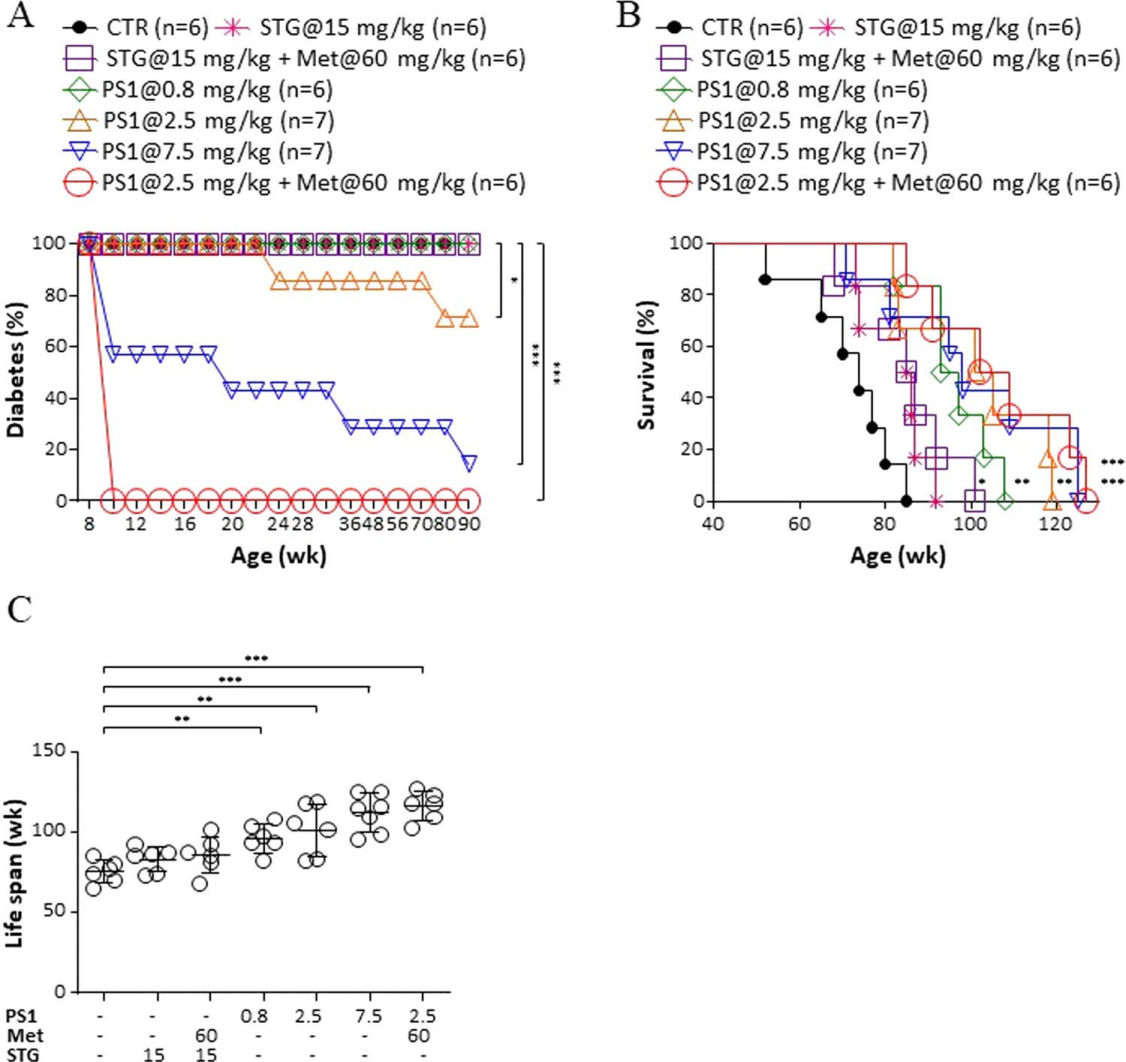
Promotion of diabetes reversal, survival rate and longevity in *db/db* mice by PS1. Diabetic incidence (**A**), survival rate (**B**), and life span (**C**) of the mice from Fig. 1A from birth to death were measured. Diabetic incidence of the mice was calculated based on their PBG over 126 mg/dL. Mouse number (*n*) is shown in parentheses. *P* (*) < 0.05, *P* (**) < 0.01 and *P* (***) < 0.001 were considered statistically significant

As far as diabetic incidence is concerned, 100% of new-onset diabetic *db/db* mice developed severe diabetes over time (CTR, Fig. 2A). Although STG per se and in combination with metformin lowered the blood glucose level of the *db/db* mice (Fig. 1A), such treatments failed to reduce diabetic incidence (STG@15 mg/kg versus STG@15 mg/kg + Met@60 mg/kg, Fig. 2A). However, PS1 at 2.5, and 7.5 mg/kg reduced diabetic incidence in *db/db* mice by 0%, 13–29%, and 43–86%, respectively (Fig. 2A). In sharp contrast, a combination of PS1 and metformin fully reversed diabetes in *db/db* mice (PS1@2.5 mg/kg + Met@60 mg/kg, Fig. 2A). We also examined survival rate and life span in different mouse groups. Sixteen-week treatment with PS1 significantly improved survival (PS1, Fig. 2B) and life span (PS1, Fig. 2C) in *db/db* mice according to dose. Furthermore, *db/db* mice treated with PS1 and metformin had better survival and life span (PS1@2.5 mg/kg + Met@60 mg/kg, Fig. 2B, C). Overall, the data indicated that the Pdia4 inhibitor, PS1, alone and in combination, could treat and reverse diabetes.

### PS1 increases serum insulin and serum c-peptide and improves HOMA indices in *db/db* mice

**Fig. 3:**
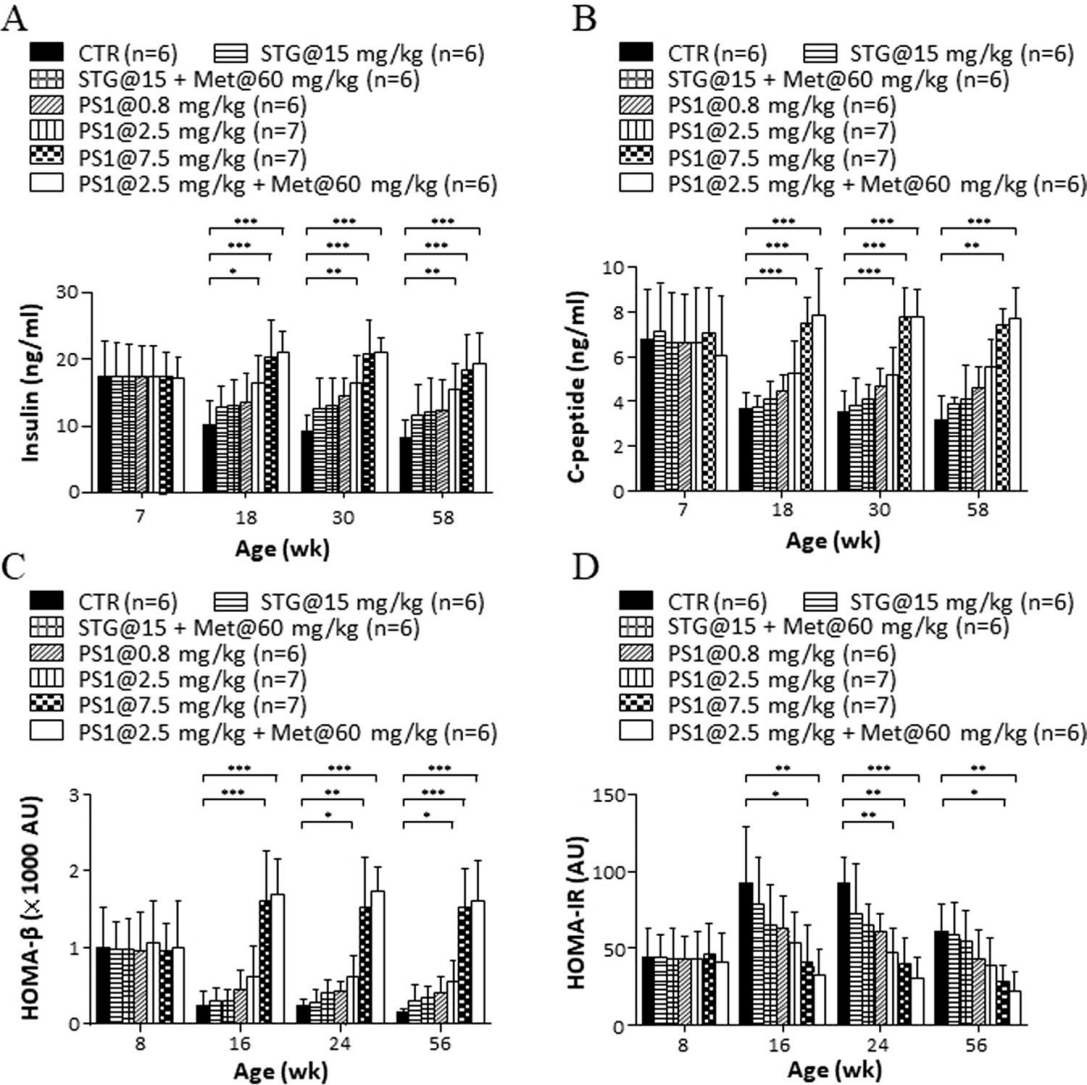
Improvement of serum insulin, c-peptide and HOMA indices in *db/db* mice by PS1. **A** The insulin concentration of the mice (Fig. 1A) was measured at the age of 7, 18, 30 and 58 weeks. **B** C-peptide of the mice (Fig. 1A) was determined at the indicated ages. HOMA-β (**C**) and (**D**) HOMA-IR of the mice (Fig. 1A) were measured at the age of 8, 16, 24 and 56 weeks. Mouse number (*n*) is shown in parentheses. *P* (*) < 0.05, *P* (**) < 0.01 and *P* (***) < 0.001 were considered statistically significant

In parallel, we tested how PS1 affected the function of pancreatic islets in *db/db* mice. No difference in levels of postprandial serum insulin was observed in any of the groups of mice aged 7 weeks (left, Fig. 3A). Levels of postmeal serum insulin in control *db/db* mice gradually decreased over time (CTR, 7–58 weeks, Fig. 3A). However, mice given STG alone and in combination with metformin had slightly more postmeal serum insulin than control mice (STG@15 mg/kg versus STG@15 mg/kg + Met@60 mg/kg, 18–58 weeks, Fig. 3A). In contrast, PS1 dose-dependently elevated the levels of postmeal serum insulin in *db/db* mice (PS1 at 0.8, 2.5 and 7.5 mg/kg, 18–58 weeks, Fig. 3A). Since c-peptide is a predictor of β-cell function, next, the effect of PS1 on the levels of c-peptide in *db/db* mice was examined. There was no difference in the levels of postprandial serum c-peptide in any of the groups of mice aged 7 weeks (7 weeks, Fig. 3B). Levels of serum c-peptide in control *db/db* mice gradually decreased over time (CTR, 7–58 weeks, Fig. 3B). However, mice given STG per se and in combination with metformin slightly increased the levels of serum c-peptide in *db/db* mice over time (STG@15 mg/kg versus STG@15 mg/kg + Met@60 mg/kg, 18–58 weeks, Fig. 3B). In contrast, PS1 per se and in combination with metformin dose-dependently augmented the levels of serum c-peptide in *db/db* mice (PS1 at 0.8, 2.5 and 7.5 mg/kg versus PS1@2.5 mg/kg + Met@60 mg/kg, 18–58 weeks, Fig. 3B). Since HOMA indices are useful markers of β-cell function and insulin resistance, next, we checked the effect of PS1 on the HOMA-β and HOMA-IR indices in diabetic *db/db* mice. There was no difference in the HOMA-β index in any group of *db/db* mice at 8 weeks (Fig. 3C). However, the HOMA-β index in *db/db* mice dramatically declined over time (CTR, Fig. 3C). STG per se and in combination with metformin modestly reduced this decline in *db/db* mice (STG@15 mg/kg versus STG@15 mg/kg + Met@60 mg/kg, 16–56 weeks, Fig. 3C). In remarkable contrast, PS1 per se and in combination with metformin significantly increased the HOMA-β index in *db/db* mice according to dose (PS1 at 0.8, 2.5 and 7.5 mg/kg versus PS1@2.5 mg/kg + Met@60 mg/kg, 16—56 weeks, Fig. 3C). In agreement, no difference in the HOMA-IR index was perceived in any group of 8-week-old *db/db* mice (Fig. 3D). However, the HOMA-IR index in *db/db* mice gradually increased over time (CTR, Fig. 3D). STG per se and in combination with metformin moderately reduced this increase in *db/db* mice (STG@15 mg/kg versus STG@15 mg/kg + Met@60 mg/kg, 16–56 weeks, Fig. 3D). PS1 per se and in combination with metformin significantly reduced the HOMA-IR index in *db/db* mice in a dose-dependent fashion (PS1@0.8, 2.5 and 7.5 mg/kg versus PS1@2.5 + Met@60 mg/kg, 16—56 weeks, Fig. 3D). As a result, the HOMA-IR index in each group of mice was inversely proportional to the HOMA-β index (Fig. 3C, D). Collectively, the data showed that PS1 ameliorated the function of the pancreatic islets.

### PS1 reduces islet atrophy, islet ROS, and serum ROS in *db/db* mice

**Fig. 4:**
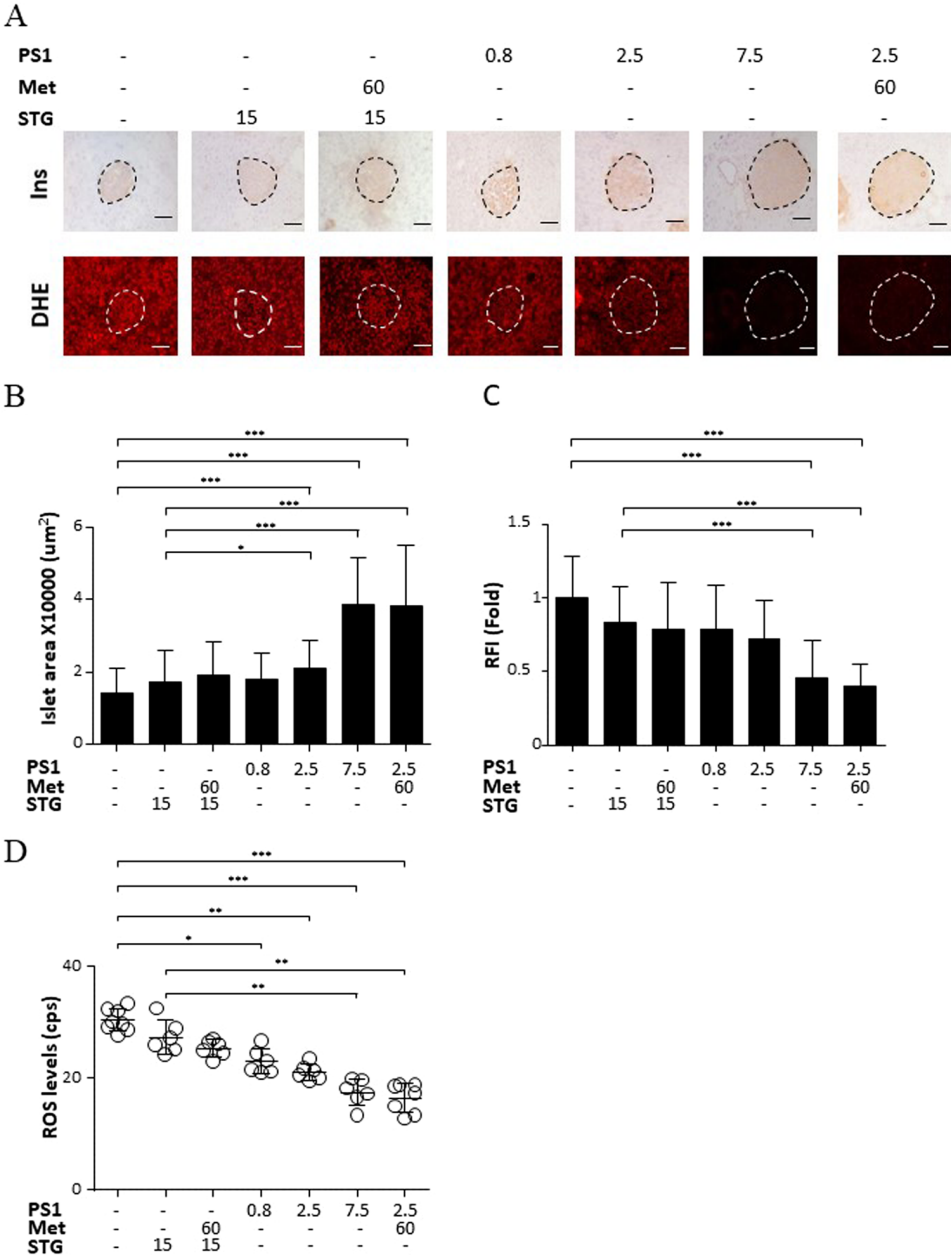
Reduction of islet atrophy, islet ROS, and serum ROS in *db/db* mice by PS1. **A** The pancreata of the mice (Fig. 1A) at the age of 18 weeks were fixed and stained with anti-insulin antibody (top) and DHE (bottom). Representative images were photographed. Scale bar: 50 µm. Islet area (μm^2^) (**B**) and relative fluorescence intensity (RFI) (**C**) of the pancreatic islets of the mice **A** were quantified and re-plotted into histograms. Scale bar = 100 μm. The dashed circles show islet regions. **D** Serum ROS of the mice (Fig. 1A) was measured at the age of 18 weeks. The number of mice (*n*) is indicated in parentheses. *P* (*) < 0.05, *P* (**) < 0.01 and *P* (***) < 0.001 were considered statistically significant

We also looked at the effect of PS1 on the architecture of pancreatic islets in *db/db* mice. Control *db/db* mice, aged 18 weeks, had smaller islet size than those treated with STG alone or in combination with metformin (STG@15 mg/kg versus STG@15 mg/kg + Met@60 mg/kg, Ins, Fig. 4A, B). Of note, *db/db* mice treated with PS1 per se and in combination with metformin further increased the islet size (PS1 at 0.8, 2.5 and 7.5 mg/kg versus PS1@2.5 + Met@60 mg/kg, Ins, Fig. 4A, B). This increase seemed to be dose-dependent. The data suggested that PS1 reduced the islet atrophy in a dose-dependent fashion. Consistently, ROS content in the islets of *db/db* mice treated with PS1 and a combination of PS1 plus metformin was inversely proportional to their islet size as evidenced by the signals of DHE staining (DHE, Fig. 4A, C). The data on the ROS content of the mouse islets were in good agreement with the data on serum ROS of *db/db* mice (Fig. 4D).

The overall data demonstrated that PS1 reduced islet atrophy and elevated the ROS level of the islets and sera in *db/db* mice.

### PS1 reduces cell death rather than cell proliferation in the islets of *db/db* mice

**Fig. 5:**
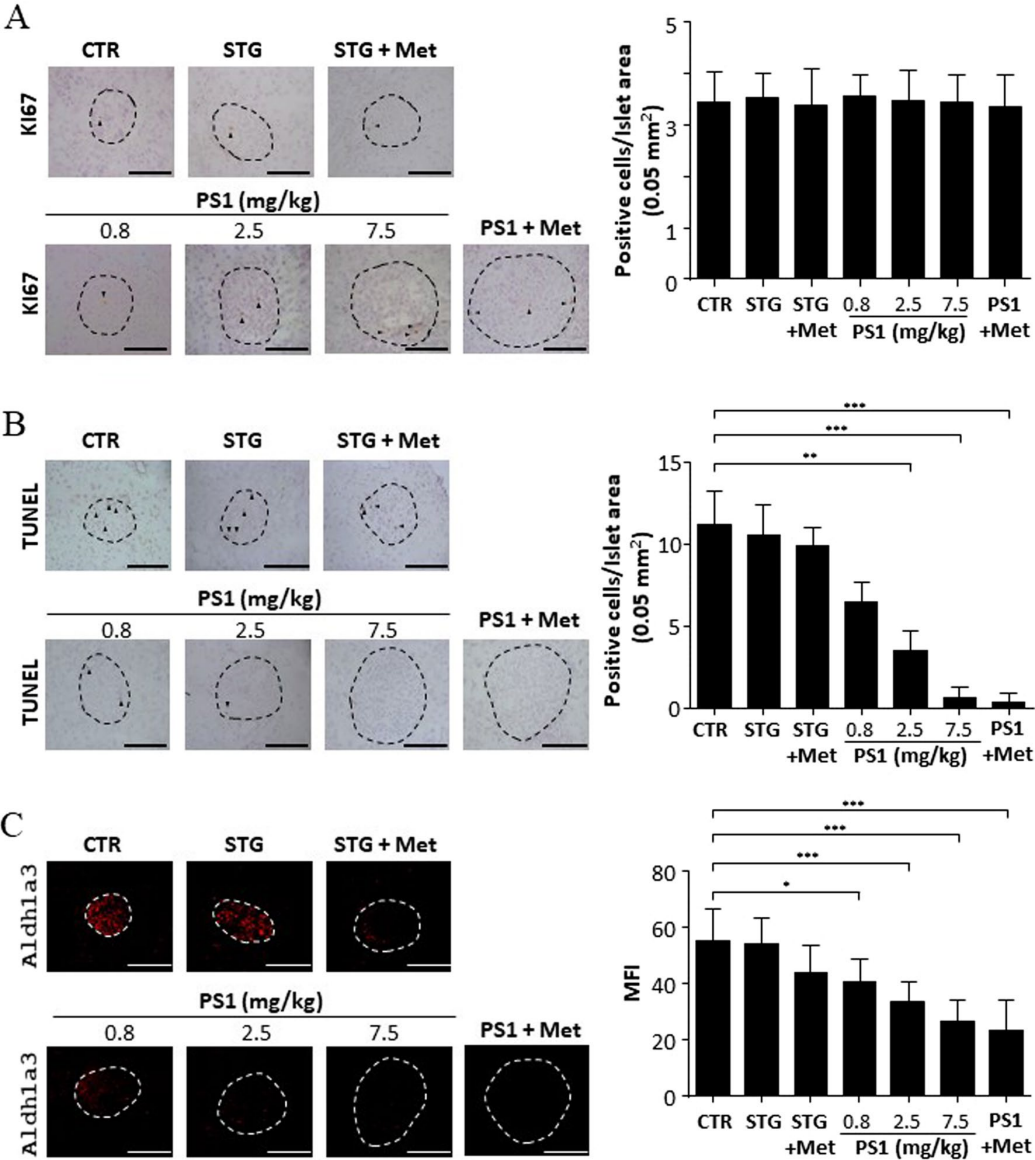
Decrease in cell death and Aldh1a3 expression in the pancreatic islets of *db/db* mice by PS1. **A**, **B** The pancreata of the mice (Fig. 1A) were fixed and stained with anti-Ki67 and TUNEL reagent. Ki67-positive cells per islet area (0.05 mm^2^) **A** were visualized in the islets of the mice (left). After quantification, the signals of each group were quantified and re-plotted into histograms (right). TUNEL-positive cells per islet area (0.05 mm^2^) **B** were visualized in the islets of the mice (left). After quantification, the signals of each group were quantified and re-plotted into histograms (right). **C** The pancreata of the mice (Fig. 1A) were fixed and stained with anti-Aldh1a3 (red). Representative images of Aldh1a3 in the islets were acquired using a confocal microscope. After quantification, the mean fluorescence intensity (MFI) of each group was quantified and re-plotted into histograms (right). The dashed circles show islet regions and the black arrowheads point out Ki67^+^ or TUNEL^+^ cells. Mouse number (*n*) is shown in parentheses. *P* (*) < 0.05, *P* (**) < 0.01 and *P* (***) < 0.001 are considered statistically significant

To probe the mechanism by which PS1 protected against β-cell failure, we first studied the action of PS1 on β-cell proliferation and demise using Ki67 staining and TUNEL assays, respectively. There was no statistical difference in the proliferation of cells of the islets among control *db/db* and the mice treated with PS1 and other drugs based on the IHC staining with the antibody against Ki67, a cell proliferation marker (Fig. 5A). However, control *db/db* mice had a comparable number of TUNEL-positive islet cells, primarily dead islet cells, as the age-matched mice treated with STG alone and in combination with metformin (STG@15 mg/kg versus STG@15 mg/kg + Met@60 mg/kg, Fig. 5B). In remarkable contrast, treatment with PS1 per se or in combination with metformin significantly diminished the number of TUNEL-positive islet cells in *db/db* mice (PS1 at 0.8, 2.5 and 7.5 mg/kg versus PS1@2.5 mg/kg + Met@60 mg/kg, Fig. 5B). Furthermore, the diminishment of TUNEL-positive islet cells in the mouse pancreata by PS1 seemed to be dose-dependent. We also explored the effect of PS1 on the expression of Aldh1a3, an indicator of β-cell dedifferentiation. PS1 dose-dependently decreased the expression level of Aldh1a3 in mouse islets (Fig. 5C). To sum up, both types of assays suggested that PS1 was able to reduce cell death but not cell proliferation in pancreatic islets of *db/db* mice.

### PS1 decreases cell death and increases insulin secretion in β-cells

**Fig. 6:**
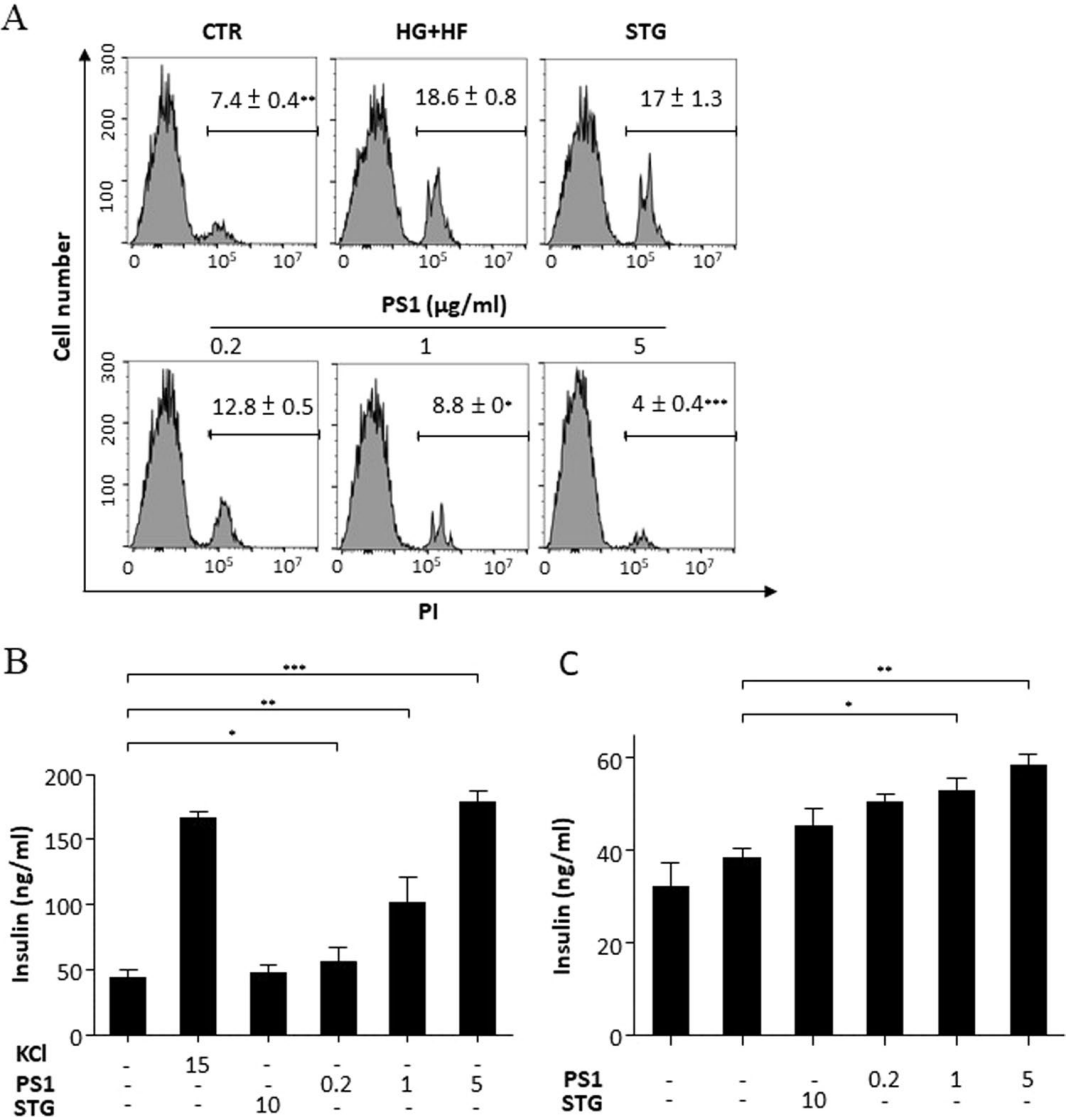
Down-regulation of cell death and up-regulation of insulin secretion in Min6 cells by PS1. **A** Min6 cells were treated with 5 mM (CTR) or 30 mM glucose with PBS (HG), STG (10 µg/mL) and PS1 (0.2 µg/mL, 1 µg/mL, and 5 µg/mL). The cells were stained with propidium iodide (PI) and analyzed with flow cytometry. The percentage of PI-positive cells from 3 experiments was analyzed and is expressed as mean ± SD. **B** Min6 cells were stimulated with DMEM medium or the medium containing KCl (30 mM), STG (10 µg/mL) and PS1 (0.2 µg/mL, 1 µg/mL, and 5 µg/mL). The insulin level of their supernatants was quantified and replotted into histograms. The data from 3 experiments are expressed as mean ± SD. **C** Min6 cells were pre-treated with DMEM medium containing 30 mM. After washing, the cells were treated with high glucose (16.7 mM), STG (10 µg/mL) and PS1 (0.2 µg/mL, 1 µg/mL, and 5 µg/mL). The insulin level of their supernatants was quantified and replotted into histograms. The data from 3 experiments are expressed as mean ± SD. *P* (*) < 0.05, *P* (**) < 0.01 and *P* (***) < 0.001 were considered statistically significant

To probe the action of PS1 in β-cells, we investigated the impact of PS1 on cell demise and function in Min6 cells, a murine β-cell line, in response to high glucose. Min6 cells were pre-incubated with cell medium, and the medium was supplemented with STG (10 μg/mL) and PS1 (0.2, 1, and 5 μg/mL) in the presence of 5 mM or 30 mM glucose. Flow cytometric data showed that control Min6 cells grown in the medium containing 5 mM glucose, had a basal level of cell death, 7.4% of PI-positive cells (CTR, Fig. 6A). Glucose at 30 mM increased cell death to 18.6% (HG, Fig. 6). STG failed to lower cell death (17%) (STG, Fig. 6A). In contrast, PS1 at 0.2 μg/mL, 1 μg/mL, and 5 μg/mL reduced cell death to 12.8%, 8.8%, and 4%, respectively (PS1, Fig. 6A). Remarkably, PS1 at 5 μg/mL even reduced this death in Min6 cells by 3.4% (from 7.4 to 4%). The data demonstrated that PS1 dose-dependently rescued Min6 cells from cell death as a result of high glucose. However, STG failed to rescue Min6 cells from cell death under the same conditions.

We also examined the effect of PS1 on insulin release in Min6 cells. First, we tested the insulinotropic effect of PS1 in Min6 cells. As expected, there was a basal level of insulin in the supernatant of Min6 cells (CTR, Fig. 6B). Potassium chloride, a positive control, elevated the level of insulin in the supernatant of Min6 cells (KCL, Fig. 6B). STG did not alter the level of insulin in the supernatant of Min6 cells (STG, Fig. 6B). However, PS1 dose-dependently up-regulated the level of insulin in the supernatant of Min6 cells (PS1, Fig. 6B). In parallel, we also treated Min6 cells with 30 mM glucose with or without PS1. As expected, high glucose compromised the glucose stimulated insulin secretion (GSIS) in Min6 cells (CTR, Fig. 6C), However, STG failed to improve the GSIS in Min6 cells (STG, Fig. 6C). In remarkable contrast, PS1 dose-dependently improved the GSIS in Min6 cells (PS1, Fig. 6C). The data suggest that PS1 protected against β-cell dysfunction and death.

### PS1 down-regulates ROS production via the Pdia4/Ndufs3 and Pdia4/p22 axes in β cells

**Fig. 7:**
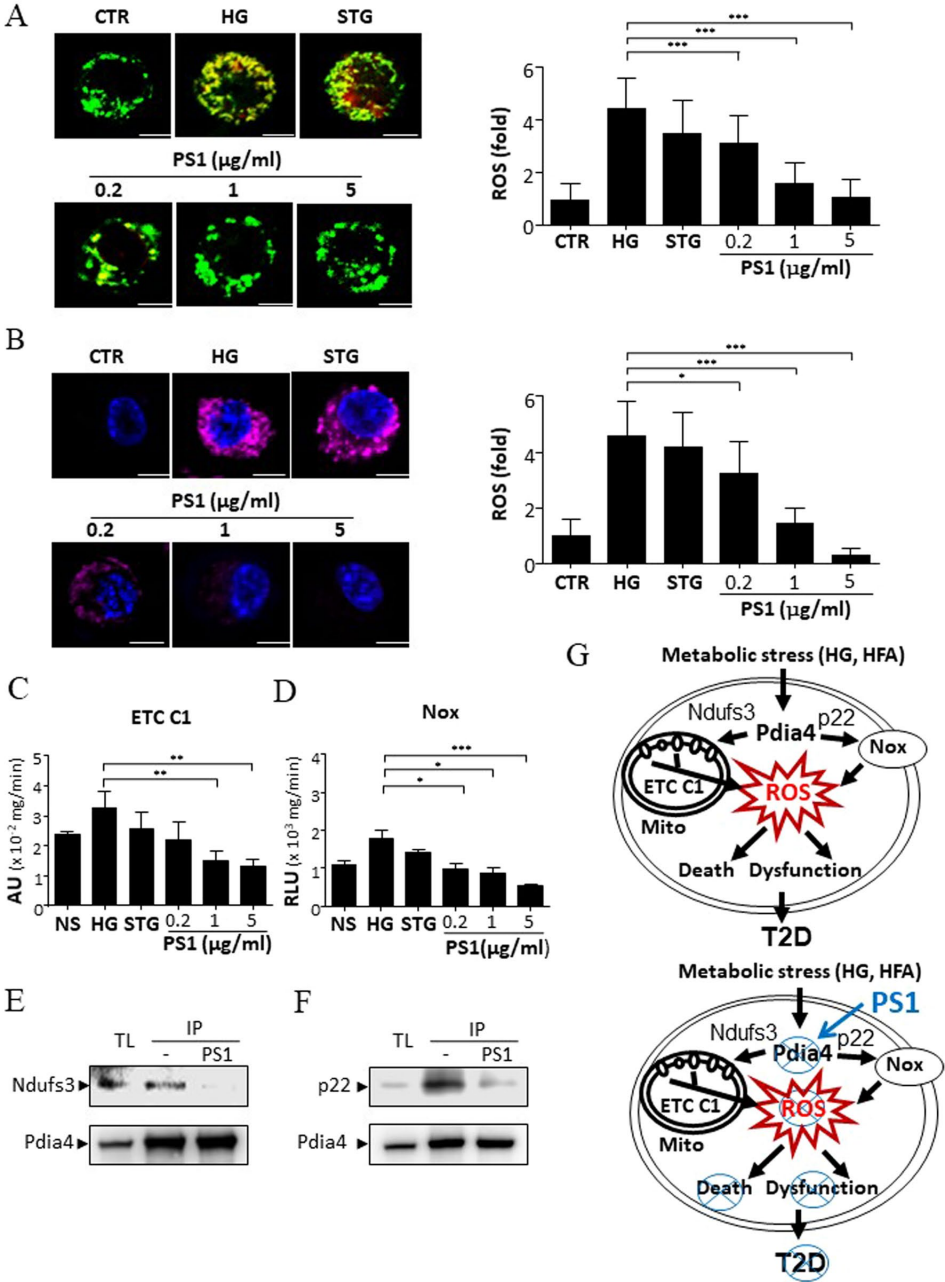
Inhibition of the Pdia4/ETC C1 and Pdia4/Nox pathways in Min6 cells by PS1. (A-B) Min6 cells were treated with 5 mM glucose (CTR) or 30 mM glucose with PBS (HG), STG (15 μg/mL), and PS1 (0.2 µg/mL, 1 µg/mL, and 5 µg/mL). The cells were then incubated with MitoGreen plus MitoSOX (**A**) and Hoechst 33,342 (Ho) plus CellROX (**B**) in the presence of glucose at 5 mM (CTR) and 30 mM (HG). The cells were visualized (left) and quantified (right). Scale bar: 5 µm. **C** The ETC C1 (left) activity of mitochondria isolated from control and PS1-treated Min6 cells **A** was measured using MitoCheck assays. **D** The Nox activity of the membrane fraction of control and PS1-treated Min6 cells **A** was measured using luminometric assays. Min6 cells that expressed Flag-Pdia4 plus Myc/Flag-tagged Ndufs3 (**E**) or p22 (**F**) were treated with PS1 (5 μg/mL) for 30 min. The cells were lysed and precipitated using anti-Pdia4 antibodies and protein G beads. Their total lysates (TL) and immunoprecipitates (IP) were analyzed with immunoblots using anti-Flag antibody. *P* (*) < 0.05, *P* (**) < 0.01 and *P* (***) < 0.001 were considered statistically significant. **G** Schema outlining the regulation of the Pdia4/Ndufs3 and Pdia4/p22 pathways. The intermolecular association of Pdia4 with Ndufs3 and p22phox increases the activity of ETC C1 and Nox. As a result, the excess ROS induces β-cell pathology and diabetes (top). On the other hand, Pdia4 inhibition by PS1 can disrupt the interplay of Pdia4 and Ndufs3 or p22, resulting in declined ROS production, β-cell failure and diabetes (bottom)

To investigate whether PS1 exerted its action through ROS pathways, we first analyzed the amount of ROS in the mitochondria and cytosol of Min6 cells. Min6 cells had a basal level of mitochondrial ROS (CTR, Fig. 7A). High glucose augmented mitochondrial ROS in Min6 cells (HG, Fig. 7A). STG failed to diminish mitochondrial ROS in Min6 cells (STG, Fig. 7A). In sharp contrast, PS1 dose-dependently reduced mitochondrial ROS in Min6 cells (PS1, Fig. 7A). Similarly, we found that Min6 cells had a basal level of cytosolic ROS (CTR, Fig. 7B). High glucose increased cytosolic ROS in Min6 cells (HG, Fig. 7B). STG failed to diminish cytosolic ROS in Min6 cells (STG, Fig. 7B). In sharp contrast, PS1 dose-dependently reduced cytosolic ROS in Min6 cells (PS1, Fig. 7B). The data showed that PS1 reduced ROS in β-cells.

To pinpoint the pathways that PS1 targeted, we checked the effect of PS1 on Pdia4 activity and the intermolecular interaction between Pdia4 and Ndufs3 or p22. The data showed that PS1 could inhibit the enzymatic activity of Pdia4 in Min6 cells (Figure S1) and, in turn, decreased the activity of ETC C1, Fig. 7C) and Nox (Fig. 7D). Further, immunoblotting data showed that PS1 intervened with the intermolecular interaction between Pdia4 and Ndufs3 (Fig. 7E) and that between Pdia4 and p22 (Fig. 7F). Taken together, the data suggested that PS1 down-regulated production of mitochondrial and cytosolic ROS via the Pdia4/Ndufs3 and Pdia4/p22 pathways in β-cells.

A schematic model describing the pharmacological function and mechanism of PS1 is shown in Fig. 7G. Under hyperglycemia, nutrient overload up-modulated Pdia4 expression. This up-modulation increased the activity of Ndufs3 and p22 through intermolecular interplay and thus escalated the generation of ROS in β-cells. Eventually, the excessive ROS caused β-cell dysfunction and death and, subsequently, diabetes (Fig. 7G). On the other hand, PS1 reversed diabetes through reduced ROS production and β-cell pathology in diabetic animals (Fig. 7G). The data also unveiled the pharmacological action and mechanism of PS1 in β-cell pathology and diabetes.

## Discussion

The *db/db* mouse, whose leptin receptor is mutated, is a commonly used model of type 2 diabetes. Our previous seminal paper showed the regulation of β-cell pathology and diabetes by Pdia4 in *db/db* mice [2]. This regulation was through the association of Pdia4 with Ndufs3 and p22, resulting in the activation of ETC C1 and Nox pathways and, hence, ROS production in β-cells [2]. One hit, 2-β-D-glucopyranosyloxy1-hydroxytrideca 5,7,9,11-tetrayne, was shown to inhibit Pdia4 activity with an IC_50_ of 358 μM. Consequently, it suppressed diabetes progression in *db/db* mice [2]. In this study, monotherapy and combinational therapy of PS1, a Pdia4-based drug candidate with an IC_50_ of 4 μM, could lessen β-cell failure and, in turn, diabetes development in *db/db* mice. Indeed, PS1 was more potent than the aforesaid hit. Mechanistically, PS1 down-regulated the ROS-generating pathways by disrupting the association of Pdia4 with Ndufs3 and p22, two important players of ETC C1 and Nox 1–4, respectively (Fig. 7). More importantly, we unveiled, for the first time, the pharmacological function of PS1 in endocrine β-cells, addressed the importance, molecular basis and therapeutic potential of PS1 in pathological process of β-cells and diabetes, and provided a novel molecular mechanism through which PS1 intervened with the ROS-generating machinery, ECT C1 and Nox, leading to shutting down ROS production.

Oxidative stress is known to dictate β-cell physiology and pathology and diabetes [20]. Consistently, metabolic stress has been reported to elevate oxidative stress through the production of ROS, which dampens insulin release and insulin action during diabetes [21]. Akin to the previous findings [2], PS1 inhibited Pdia4 activity (Figure S1) and, thus, abolished the interplay between Pdia4 and Ndufs3 or p22 in β-cells (Fig. 7E, F). Disruption of Pdia4 might destabilize Ndufs3 or p22 and dampened ROS-generating ETC C1 and Nox pathways as published [2]. Accordingly, PS1 also reduced the activity of ETC C1 and Nox (Fig. 7C, D). As expected, PS1 reduced ROS content in the pancreatic islets of *db/db* mice (Fig. 4A, C) and in the cytosol and mitochondria of β-cells (Fig. 7A, B). In fact, the above data are in good agreement with several reports that have stated the escalated expression and/or activity of p22 and Ndufs3 in humans and animals of diabetes [22, 23]. Since Pdia4 is not an essential gene [2, 24], its specific inhibitor, PS1, seemed to have no noticeable adverse effects. On the contrary, PS1 was able to reverse diabetes, increase survival and prolong the longevity in *db/db* mice (Fig. 2). Overall, we believe the data presented herein move us one step further towards clinical use of the Pdia4-based therapy for β-cell pathogenesis and diabetes.

Our previous publication demonstrated that Pdia4 knockout reduced cell death but not cell proliferation in the β-cells of *db/db* mice [2]. As a result, Pdia4 ablation accumulated functional β-cells and, in turn, reversed diabetes in diabetic animals. In good agreement with the publication on Pdia4 ablation, 16-week administration of PS1 alone and together with metformin normalized diabetes in *db/db* mice as shown by blood glucose, Hb_A1c_, GTT, diabetic incidence, and water consumption (Figs. 1, 2, S2B). Of note, PS1 per se and in combination with metformin increased longevity of *db/db* mice by 29 and 34 weeks, respectively (Fig. 2C). The data suggested a good efficacy of PS1 in diabetes. Strikingly, the reversal rate of diabetes for PS1 per se and in combination with metformin was 86% and 100% in *db/db* mice even after 68-week drug withdrawal (Fig. 2A). Consequently, we found that PS1 reduced cell death but not cell proliferation in the β-cells of *db/db* mice (Fig. 5A, B). The data clearly demonstrated that PS1 diminished β-cell demise in diabetic animals. Flow cytometric data showed that in response to high glucose, PS1 dose-dependently protected against cell death in β-cells (Fig. 6A). Meanwhile, PS1 reduced the decline of c-peptide and insulin in the sera of *db/db* mice (Fig. 3A, B). Moreover, PS1 increased the HOMA-β index and decreased the HOMA-IR index in *db/db* mice (Fig. 3C, D). ELISA data also showed that PS1 enhanced insulin release in β-cells (Fig. 6B). More importantly, PS1 could reduce the deterioration of insulin release in β-cells pre-treated with high glucose (Fig. 6C). Overall, the data suggested that PS1 ameliorated β-cell function in diabetic animals. Likewise, PS1 decreased the expression level of Aldh1a3 (Fig. 5C), which was reported to participate in β-cell failure [25, 26]. The above data demonstrate the importance and efficacy of PS1, which targets Pdia4, to preserve functional β-cells in diabetes therapy.

In this study, *db/db* mice developed diabetes, and also obesity. Accumulating evidence supports a causal relationship between obesity and ER stress in adipose tissue. Pdia4 has been reported to be a novel ER chaperone implicated in β-cell pathogenesis in diabetes [2]. However, the role of Pdia4 in obesity progression remains poorly understood. One report stated that Pdia4 regulated adipocytes by down-regulating adiponectin [27]. Consistently, serum Pdia4 was shown to be related to obesity, insulin sensitivity, and diabetes [28]. The adiponectin/leptin ratio was proposed as a functional marker of fat inflammation in human patients of diabetes [29]. In this sense, it is necessary to verify whether Pdia4 affects this ratio in *db/db* mice. In addition, moderate islet inflammation was thought to contribute to β-cell failure in type 2 diabetes. Alternatively activated macrophages facilitated a loss of β-cell identity in *db/db* mice [30]. Given the relationship between Pdia4 and obesity, it should be noted that other factors like aquaporins, a central player in fat metabolism, could be involved in the Pdia4 pathways [31]. Given the importance of Pdia4 for obesity, it will be absolutely necessary to further explore the function and mechanism of PS1 in obesity.

## Supplementary Information

Below is the link to the electronic supplementary material.

Supplementary file1 (PDF 309 kb)

## Data Availability

The raw data supporting the conclusions of this manuscript are included in this manuscript and Supplementary Information.

## Abbreviations

CM-H2DCFDA: Chloromethyl-2′,7′-dichlorodihydrofluorescein diacetate
DAB: Diaminobenzidine tetrahydrochloride
DHE: Dihydroethidium
ELISA: Enzyme-linked immunosorbent assay
ER: Endoplasmic reticulum
Ero1: ER oxidoreductin 1
ETC C1: Electron transport chain complex 1
FBG: Fasting blood glucose
GSIS: Glucose stimulated insulin secretion
GTT: Glucose tolerance test
Hb_A1c_: Hemoglobin A1c
HOMA-β and HOMA-IR: Homeostatic model assessment for β-cell function and insulin resistance
IC_50_: Half maximal inhibitory concentration
IHC: Immunohistochemical
Nox: NADPH oxidase
OCT: Optimal cutting temperature
PBG: Postprandial blood glucose
Pdia4: Protein disulfide isomerase a4
PI: Propidium iodide
ROS: Reactive oxygen species
STG: Sitagliptin
TUNEL: Terminal deoxynucleotidyl transferase dUTP nick end labeling
FDA: Food and Drug Administration of the United States
