# Supplementary material for: Pharmacological and mechanistic study of PS1, a Pdia4 inhibitor, in β-cell pathogenesis and diabetes in db/db mice
Source: Cell Mol Life Sci. 2023 Mar 19;80(4):101. doi: 10.1007/s00018-022-04677-5 (PMC10025235; doi:10.1007/s00018-022-04677-5)
Supplement: Supplementary file 1 — Supplementary file1 (PDF 309 kb) [file 18_2022_4677_MOESM1_ESM.pdf]

**Pharmacological and mechanistic study of PS1, a Pdia4 inhibitor, in β-cell pathogenesis and diabetes in db/db mice**

**Supplementary figures**

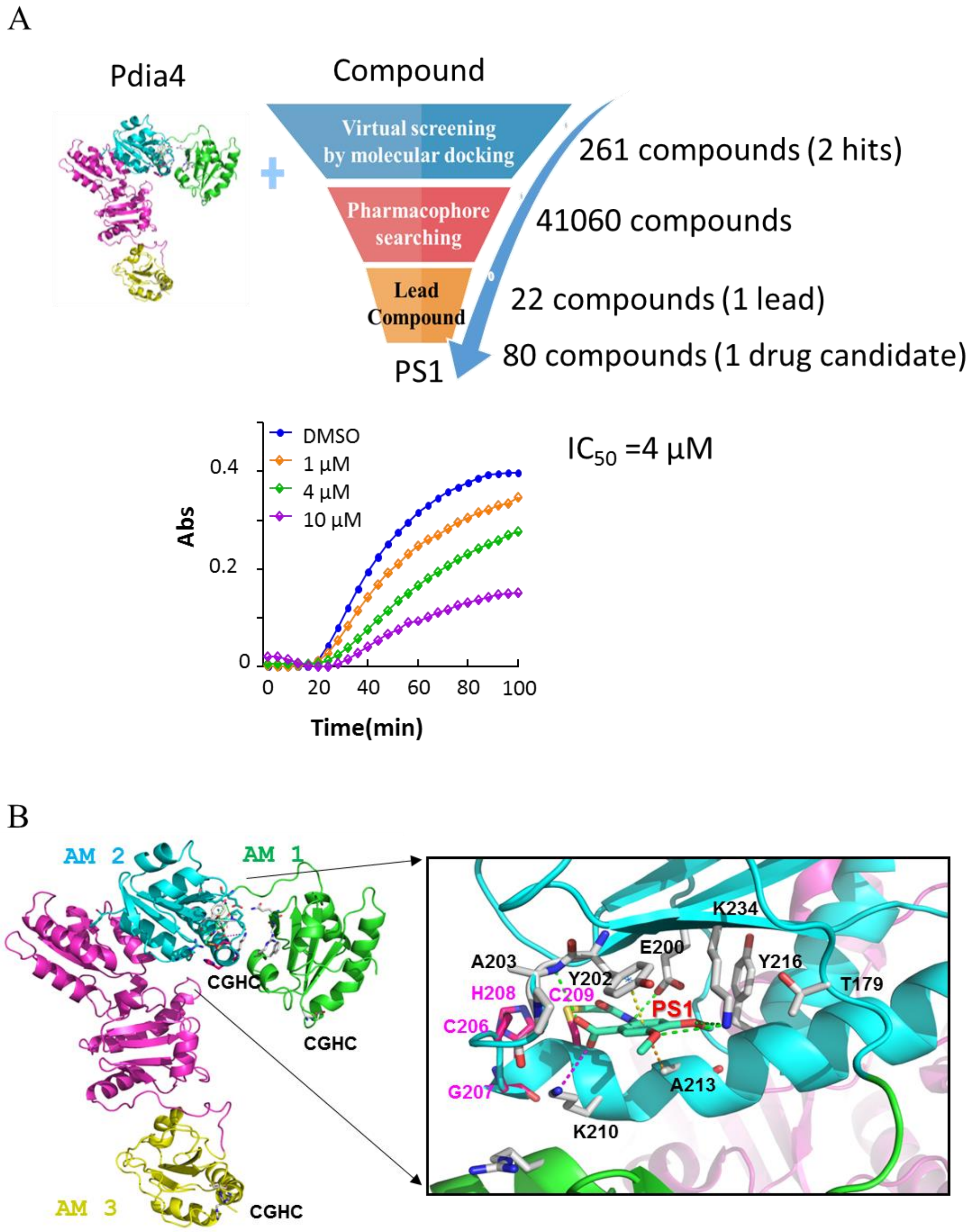


**Figure S1. Identification of PS1 as a potent Pdia4 inhibitor using molecular docking, total chemical synthesis, and Pdia4 bioassays.** (A) Pdia4 bioassays and virtual screening based on the homology model of Pdia4 were applied to search hits, leads

and/or drug candidates out of 261 plant compounds using molecular docking that contained three CGHC motifs. The two best hits were identified, Next, based on the pharmacophore features of the two hits and the binding pocket of Pdia4, 22 compounds were selected from the ZINC database (41060 compounds) using the virtual screening and their$IC_{50}$ values were measured in an attempt to determine the core structure of leads. One lead out of the 22 compounds was determined as the active core structure based on Pdia4 assays, followed by a series of lead optimization (80 compounds). Consequently, one of drug candidates, PS1, stood out as a potential compound (top). The $IC_{50}$ value of PS1 was determined using Pdia4 bioassays (bottom). (B) Molecular docking indicates the interaction between PS1 and active motifs (AMs) of Pdia4. AM 1, 2 and 3 represent the first, second and third CGHC domains. The sulfur, nitrogen and oxygen atoms are shown in yellow, blue, and red, respectively. The hydrogen bond and hydrophobic interaction between PS1 (light green) and the amino acid residues (gray) of Pdia4 model are shown by the green and yellow dashed lines, respectively.

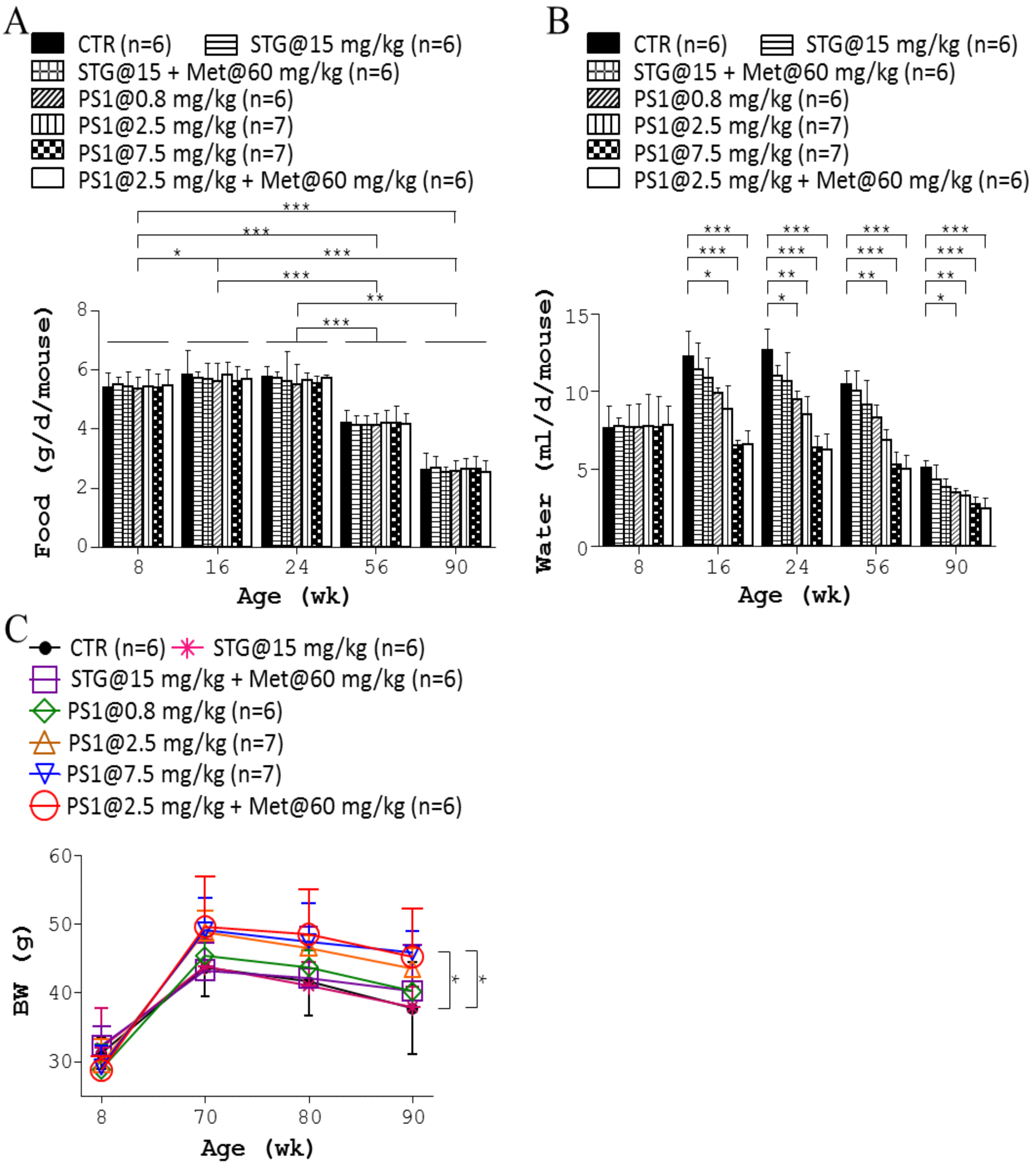


**Figure S2. Effect of PS1 on food intake, water consumption and body weight in db/db mice.**

(A-C) The mice from Figure 1A were monitored for their consumption of food (A) and water (B) and body weight (BW, C) from birth to 90 weeks. The number of mice (n) is indicated in parentheses. *P* (*) < 0.05, *P* (**) < 0.01 and *P* (***) < 0.001 are considered statistically significant.
